# Protective role of cytoplasmic p21Cip1/Waf1 in apoptosis of CDK4/6 inhibitor‐induced senescence in breast cancer cells

**DOI:** 10.1002/cam4.4410

**Published:** 2021-11-11

**Authors:** Irna D. Kartika, Hitoshi Kotani, Yuichi Iida, Akira Koyanagi, Ryosuke Tanino, Mamoru Harada

**Affiliations:** ^1^ Department of Immunology Shimane University Faculty of Medicine Shimane Japan; ^2^ Department of Clinical Pathology Faculty of Medicine University of Muslim Indonesia Sulawesi Indonesia; ^3^ Division of Medical Oncology & Respiratory Medicine Department of Internal Medicine Shimane University Faculty of Medicine Shimane Japan

**Keywords:** abemaciclib, ABT‐263, breast cancer, p21, senescence

## Abstract

Inhibition of CDK4/6 slows the cell cycle and induces senescence in breast cancer cells. However, senescent cancer cells promote invasion and metastasis. Several drugs reportedly target senescent cells, including ABT‐263 (navitoclax). We examined the effects of the CDK4/6 inhibitor abemaciclib and ABT‐263 on two human breast cancer cell lines. The abemaciclib and ABT‐263 combination additively decreased the viability of MDA‐MB‐231 cells, but not MCF‐7 cells. Also, the combination therapy‐induced caspase‐dependent apoptosis in MDA‐MB‐231 cells. Combination therapy with abemaciclib and ABT‐737, an ABT‐263 analog, significantly suppressed the in vivo growth of MDA‐MB‐231 with transient body‐weight loss. Given that p16^Ink4a^ and p21^Cip1/Waf1^ are key factors in senescence and that both cell lines were negative for p16, the role of p21 in apoptosis of treated breast cancer cells was investigated. Although abemaciclib increased the cytoplasmic p21 level in both cell lines as a hallmark of senescence, the abemaciclib and ABT‐263 combination decreased it only in MDA‐MB‐231 cells. This decrease of p21 expression was relieved by caspase inhibition, and p21 was colocalized with caspase‐3 in the cytoplasm of MDA‐MB‐231 cells. Alternatively, small interfering RNA‐mediated knockdown of p21 rendered caspase‐3‐negative MCF‐7 cells susceptible to abemaciclib and ABT‐263, as well as TNF‐related apoptosis‐inducing ligand. Furthermore, a clinical database analysis showed that p21^high^ breast cancer patients had a poorer prognosis compared to p21^low^ patients. These results suggest that cytoplasmic p21 plays a protective role in apoptosis of CDK4/6 inhibitor‐induced senescent breast cancer cells.

## Graphical abstract

Colocalization of p21 and caspase‐3 in treated MDA‐MB‐231 breast cancer cells.

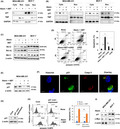

## 1 INTRODUCTION

Breast cancer is the most common type of cancer in women worldwide.^1^ Chemotherapy and hormone therapy are the principal treatment modalities for breast cancer, but several molecular‐targeting drugs have been developed. These include CDK 4/6 inhibitors, which were approved for the treatment of hormone receptor‐positive, HER2‐negative metastatic breast cancer.^2^ CDK4/6 inhibitors hinder the transition from the G1 to the S phase of the cell cycle with consequent growth arrest and increased expression of the cell‐cycle inhibitors, including p16^Ink4a^ and p21^Cip1/Waf1^.^3^ This state is called senescence and is induced in nonmalignant and malignant cells in response to various stresses.^4^ Anticancer agents induce senescence in cancer cells via triggering DNA damage.^5^ Senescent cells secrete several growth factors and inflammatory cytokines, known as the senescence‐associated secretory phenotype (SASP).^6^ However, as well as activating and promoting accumulation of immune cells and tumor clearance, SASP promotes cancer recurrence and metastasis.^7^

Antiapoptotic Bcl‐2 family proteins protect cancer cells from therapy‐induced apoptosis.^8^ To overcome this resistance, several inhibitors targeting these Bcl‐2 family proteins have been developed.^9^ ABT‐263 (navitoclax) is a small‐molecule inhibitor targeting Bcl‐2, Bcl‐xL, and Bcl‐w.^10^ In this regard, we reported that ABT‐263 and ABT‐737, a homologue with identical specificity,^11^ sensitize human prostate and pancreatic cancer cells to docetaxel and TNF‐related apoptosis‐inducing ligand (TRAIL), respectively.^12^, ^13^ Alternatively, several drugs, called as senolytics, have been reported to preferentially target senescent cells for death.^14^, ^15^ ABT‐263 is a representative and reportedly eliminates therapy‐induced senescent (TIS) cancer cells.^16^ Depletion of TIS cancer cells could reduce the likelihood of tumor recurrence and metastasis.

We recently reported that abemaciclib can induce senescence‐associated β‐galactosidase, a typical marker of senescence, in MDA‐MB‐231 cells.^17^ Therefore, in this study, we examined the antitumor effects of the CDK4/6 inhibitor abemaciclib and ABT‐263 using MDA‐MB‐231 and MCF‐7 human breast cancer cell lines and investigated the underlying mechanisms.

## 2 MATERIALS AND METHODS

### 2.1 Cell lines and reagents

Two human breast cancer cell lines (MDA‐MB‐231 and MCF‐7), which were kindly provided by Dr. K. Takenaga, Faculty of Medicine, Shimane University, Japan, were maintained in DMEM (Sigma‐Aldrich) supplemented with 10% fetal calf serum (Invitrogen) and 20 µg/ml gentamicin (Sigma‐Aldrich) at 37°C in a humidified atmosphere of 5% CO_2_. Abemaciclib was obtained from LKT Labs. ABT‐263 and ABT‐737 were obtained from Active Biochemicals. ABT‐199 was obtained from ChemieTek. The pan‐caspase inhibitor (z‐VAD‐FMK) was obtained from Enzo Life Sciences, and both caspase‐8 inhibitor (z‐IETD‐FMK) and caspase‐9 inhibitor (z‐LEHD‐FMK) were obtained from R&D Systems. *N*‐acetyl‐l‐cysteine (NAC) was obtained from Nacalai Tesque. Recombinant TRAIL was obtained from PeproTech.

### 2.2 Assay of cell viability

Cell viability was measured by Cell Counting Kit‐8 (CCK‐8) (DOJINDO Lab). Briefly, cells were cultured in flat‐bottomed 96‐well plates for 2 days, and 10 µl of CCK‐8 solution was added to wells. After 3 h, the plates were read at 450 nm using MULTISKAN FC (Thermo Scientific).

### 2.3 Flow cytometry

Cell death was assessed using the annexin V‐FITC Apoptosis Detection Kit (BioVision) and propidium iodide (PI). In some experiments, annexin V‐APC (BD Biosciences) was used. Caspase inhibitors (20 µM), or DMSO as a control, was added 30 min before culturing cells. Assay was done using a FACSCalibur flow cytometer (BD Biosciences).

### 2.4 Reactive oxygen species (ROS) assay

Intracellular ROS were assayed using carboxy‐H_2_DCFDA (Invitrogen). Carboxy‐H_2_DCFDA (25 µM) was added 30 min before harvesting. To inhibit ROS, NAC (5 mM) was added 1 h before culturing cells. After harvesting, cells were analyzed by flow cytometry.

### 2.5 Mitochondrial membrane potential (∆Ψm) assay

Cells were cultured in six‐well plates. MitoProbe™ DiOC_2_ ^3^ (Molecular Probes) at 50 nM was added 30 min before harvesting. Thereafter, cells were examined by flow cytometry after staining with annexin V‐APC (BD Biosciences).

### 2.6 Immunoblotting

Cells were lysed with radioimmunoprecipitation assay buffer (Fujifilm Wako Pure Chemical) containing protease/phosphatase inhibitor cocktail (Nacalai Tesque). Equal amounts of protein were transferred onto polyvinylidene difluoride membranes. After blocking, the blots were incubated with the following antibodies: anti‐p21^Cip1/Waf1^ (#2947; Cell Signaling Technology [CST]), anti‐p16^Ink4a^ (SPC‐1280; StressMarq Biosciences), anti‐γH2AX(Ser^139^) (#9718; CST), anti‐Bcl‐2 (#658701; BioLegend), anti‐Bcl‐xL (#2764; CST), anti‐Mcl‐1 (#54535; CST), anti‐survivin (#71G4B7; CST), anti‐cFLIP (ALX‐804–428; Enzo Life Sciences), anti‐TATA‐binding protein (TBP; #22006‐I‐AP; Proteintech), anti‐PARP (#46D11; CST), anti‐caspase‐3 (#9668; CST), anti‐c‐Myc (1472–1; EPT), anti‐GAPDH (#015‐25473; Fujifilm Wako Pure Chemical), and anti‐β‐actin (#622102; BioLegend). Nuclear and cytoplasmic proteins were prepared using the LysoPure™ Nuclear and Cytoplasmic Extraction Kit (Fujifilm Wako Pure Chemical). After washing, membranes were incubated with goat anti‐rabbit or horse anti‐mouse horseradish peroxidase‐conjugated secondary antibody (#7074 and #7076; CST). Protein bands were visualized using an Amersham ImageQuant™ 800 Biomolecular Imager (General Electric Company).

### 2.7 Establishment of a p21‐overexpressing cell line

MDA‐MB‐231 cells were transfected with pEB Multi‐Neo vector (Fujifilm Wako Pure Chemical), in which genes encoding p21 with IRES2‐ZsGreen (Takara Bio) were inserted, using Lipofectamine 3000 (Invitrogen) and cultured in the presence of G418. A pEB Multi‐Neo vector with *ZsGreen* gene was used as the control vector.

### 2.8 Confocal imaging

Cancer cells were cultured on round coverslips in 24‐well plates. After incubation with Hoechst 33342 (5 µg/ml) for 30 min, cells were fixed with 4% paraformaldehyde. Fixed cells were stained with anti‐p21 rabbit IgG (CST) and anti‐caspase‐3 mouse IgG (#9668S; CST), followed by Alexa 488‐conjugated anti‐rabbit antibody (#44125; CST) and Cy5‐conjugated anti‐mouse IgG (#NB76021; Novus). Coverslips were placed on slide glasses with 4 µl of Vectashield mounting medium (Vector Laboratories) and observation was done by confocal laser scanning microscopy FV1000‐D (Olympus).

### 2.9 In vivo xenograft model

Female BALB nude mice (CLEA Japan) were injected with MDA‐MB‐231 (3 × 10^6^) cells and Matrigel (Japan BD Biosciences) at a 1:1 volume ratio in 100 μl into the right mammary pad. When the tumor diameter was approximately 5–6 mm, the mice were divided into four groups. Abemaciclib (50 mg/kg) was orally administered on day 0–7 after grouping. On days 2, 4, and 6 after grouping, breast cancer‐bearing mice were administered intraperitoneally ABT‐737 (50 mg/kg). The tumor volume (mm^3^) and body weight were measured twice weekly. The tumor volume was calculated as follows: tumor volume = (length × width^2^) ÷ 2. The experimental protocol was approved by the Committee on the Ethics of Animal Experiments of the Shimane University Faculty of Medicine (IZ3‐74).

### 2.10 Transfection of siRNAs

siRNA transfection was performed using Lipofectamine RNAiMAX (Life Technologies). The following siRNAs were used: p21(I) siRNA (#6456; CST), p21(II) siRNA (#6558; CST), and control siRNA (#6568; CST). Two days after siRNA transfection, transfected cancer cells were used for experiments.

### 2.11 Clinical data analysis

The Kaplan–Meier plotter^18^ was used for univariate analysis of survival time according to CDKN1A gene expression in breast cancer. Version 2021 of the database was used for analysis. Outlier array data were excluded for array quality control. Patients were split into low‐ and high‐expression groups based on the best cutoff. TRGAted^19^ was used to analyze survival according to tumor p21 protein level in patients with breast invasive carcinoma. All subtypes of the TCGA‐BRCA‐L4 dataset were used in the analysis. Patients were split into low‐ and high‐expression groups based on the optimal cutoff.

### 2.12 Statistical analysis

Student's *t* test (two groups) and analysis of variance (ANOVA) with the Tukey–Kramer test (more than two groups) were used. A *p* value < 0.05 was judged statistically significant.

## 3 RESULTS

### 3.1 Antitumor effects of abemaciclib and/or ABT‐263 on breast cancer cells

**FIGURE 1:**
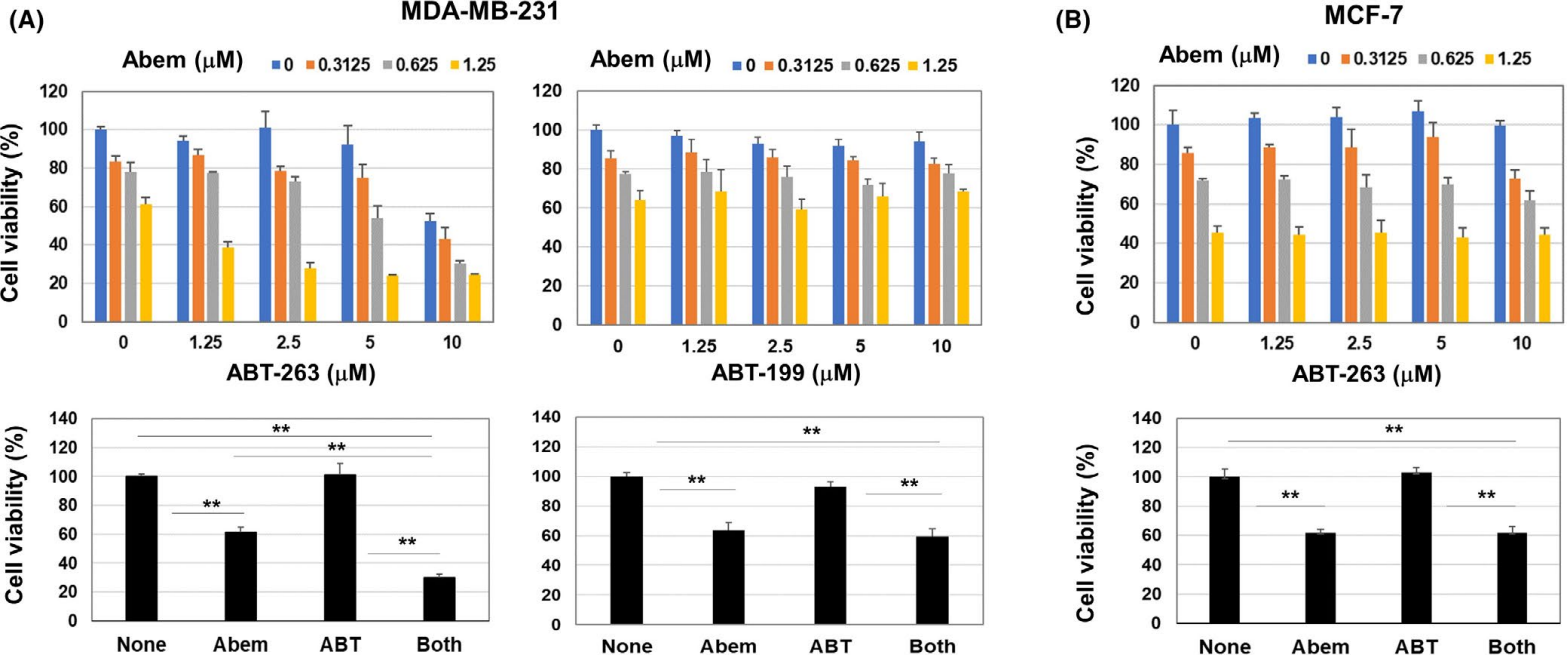
In vitro effects of abemaciclib and/or ABT‐263 on two breast cancer cell lines. (A) MDA‐MB‐231 cells were cultured with abemaciclib and ABT‐263 or ABT‐199 for 2 days. Cell viability (%) was determined by CCK‐8 assay. Data are the means ± SD of three wells. (Low) Selected results (abemaciclib [1.25 µM] and ABT‐263/ABT‐199 [2.5 µM]) are shown. **p < 0.01. (B) Similarly, MCF‐7 cells were cultured with abemaciclib and/or ABT‐263 for 2 days. (Low) Selected results (abemaciclib [1.25 µM] and ABT‐263 [2.5 µM]) are shown. Similar results were obtained in three independent experiments. **p < 0.01

The effects of abemaciclib and/or ABT‐263 on two human breast cancer cell lines were examined. Abemaciclib or ABT‐263 alone decreased the viability of MDA‐MB‐231 cells in a dose‐dependent manner, whereas ABT‐199 showed no effect on their viability (Figure 1A). Combination of abemaciclib and ABT‐263 additively decreased the viability of MDA‐MB‐231 cells, but such effect was not observed when abemaciclib was combined with ABT‐199. On the other hand, abemaciclib alone decreased MCF‐7 cell viability more effectively compared with the case of MDA‐MB‐231 cells, whereas ABT‐263 showed no effect on MCF‐7 cells (Figure 1B). Combination of abemaciclib and ABT‐263 showed no additive effect on MCF‐7 cells. Given that ABT‐199 is a Bcl‐2 inhibitor and ABT‐263 is an inhibitor against Bcl‐2, Bcl‐xL, and Bcl‐w,^9^ Bcl‐xL inhibition by ABT‐263 might contribute to the antitumor effect of the combination therapy on MDA‐MB‐231 cells.

### 3.2 Apoptosis in MDA‐MB‐231 cells treated with both abemaciclib and ABT‐263

**FIGURE 2:**
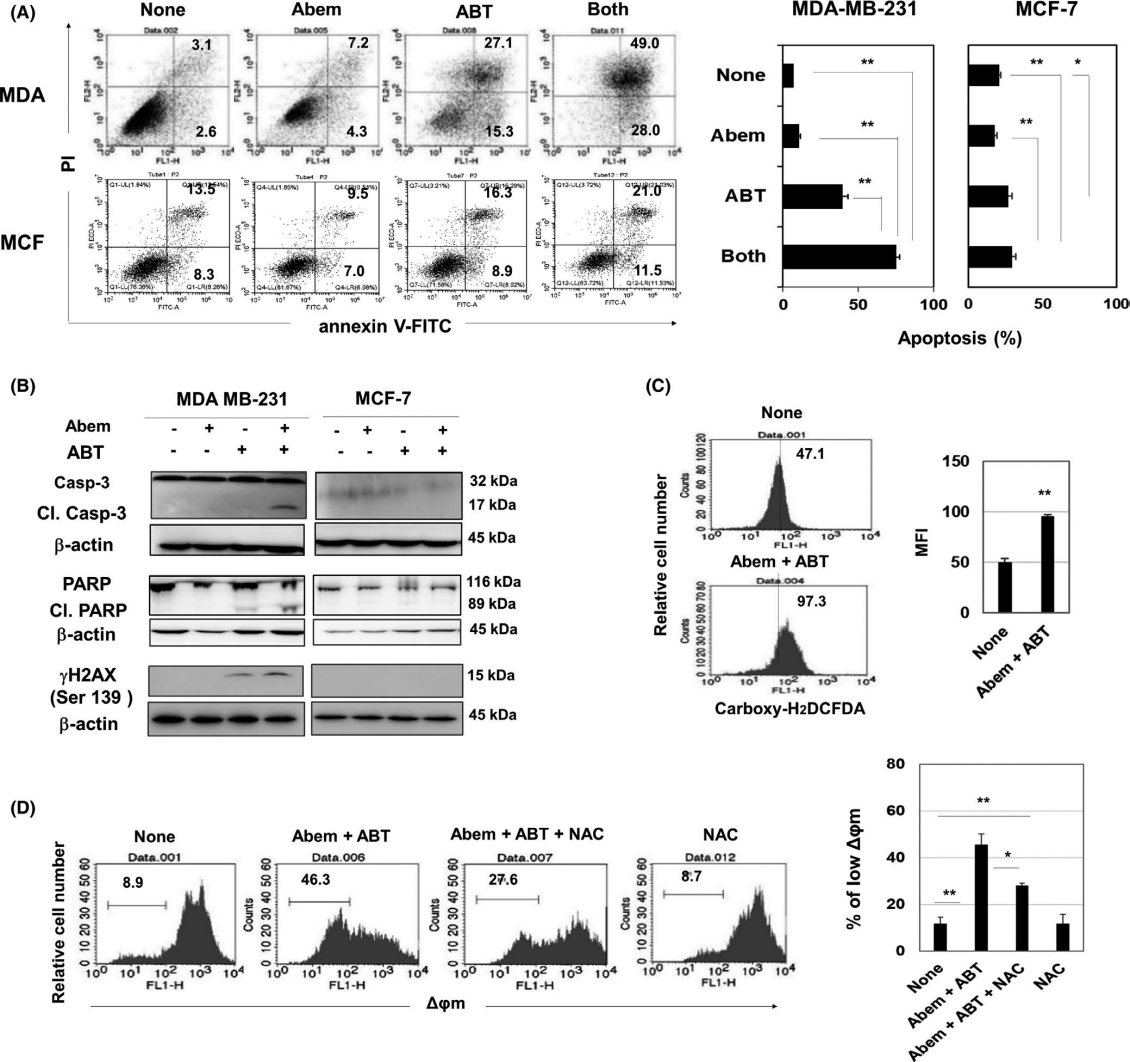
Apoptosis of MDA‐MB‐231 cells treated with abemaciclib and ABT‐263. (A) Cancer cells were cultured with abemaciclib (1.5 µM) and ABT‐263 (1.5 µM) for 2 days. Flow cytometric analysis was done after staining with annexin V‐FITC and PI. Numbers represent the proportions (Right). Data are the means ± SD of three wells are shown. *p < 0.05, **p < 0.01. (B) Lysates of cancer cells, which were cultured with abemaciclib (1.5 µM) and ABT‐263 (1.5 µM) for 2 days, were subjected to immunoblotting. (C) MDA‐MB‐231 cells were cultured with abemaciclib (1.5 µM) and ABT‐263 (1.5 µM) for 2 days. During the last 30 min, carboxy‐H_2_DCFDA was added, and harvested cells were analyzed by flow cytometry. The numbers represent the mean fluorescence intensity. **p < 0.01. (D) Similarly, treated MDA‐MB‐231 cells were analyzed by flow cytometry. NAC was added 1 h before the addition of drugs. The numbers represent the proportions. (Right) Data are the means ± SD of three wells. *p < 0.05, **p < 0.01

Given that a decrease in cell viability can reflect growth arrest or cell death, we next assayed cell death. ABT‐263 alone increased the proportions of annexin V^+^ apoptotic MDA‐MB‐231 cells, whereas the combination drastically increased them (Figure 2A). In contrast, their combination increased the proportions of annexin V^+^ MCF‐7 cells, albeit only slightly. The abemaciclib and ABT‐263 combination‐induced cleavage of caspase‐3 and PARP, as well as the induction of γH2AX expression, in MDA‐MB‐231 cells (Figure 2B). MCF‐7 cells lacked caspase‐3,^20^ and cleaved PARP and γH2AX were not expressed in treated MCF‐7 cells. The combination treatment increased the level of ROS (Figure 2C) and decreased the ∆Ψm in MDA‐MB‐231 cells (Figure 2D). The addition of NAC, a scavenger of ROS, relieved the decreased ∆Ψm in treated MDA‐MB‐231 cells.

### 3.3 Growth suppression of MDA‐MB‐231 by abemaciclib and ABT‐737 in a xenografted mouse model

**FIGURE 3:**
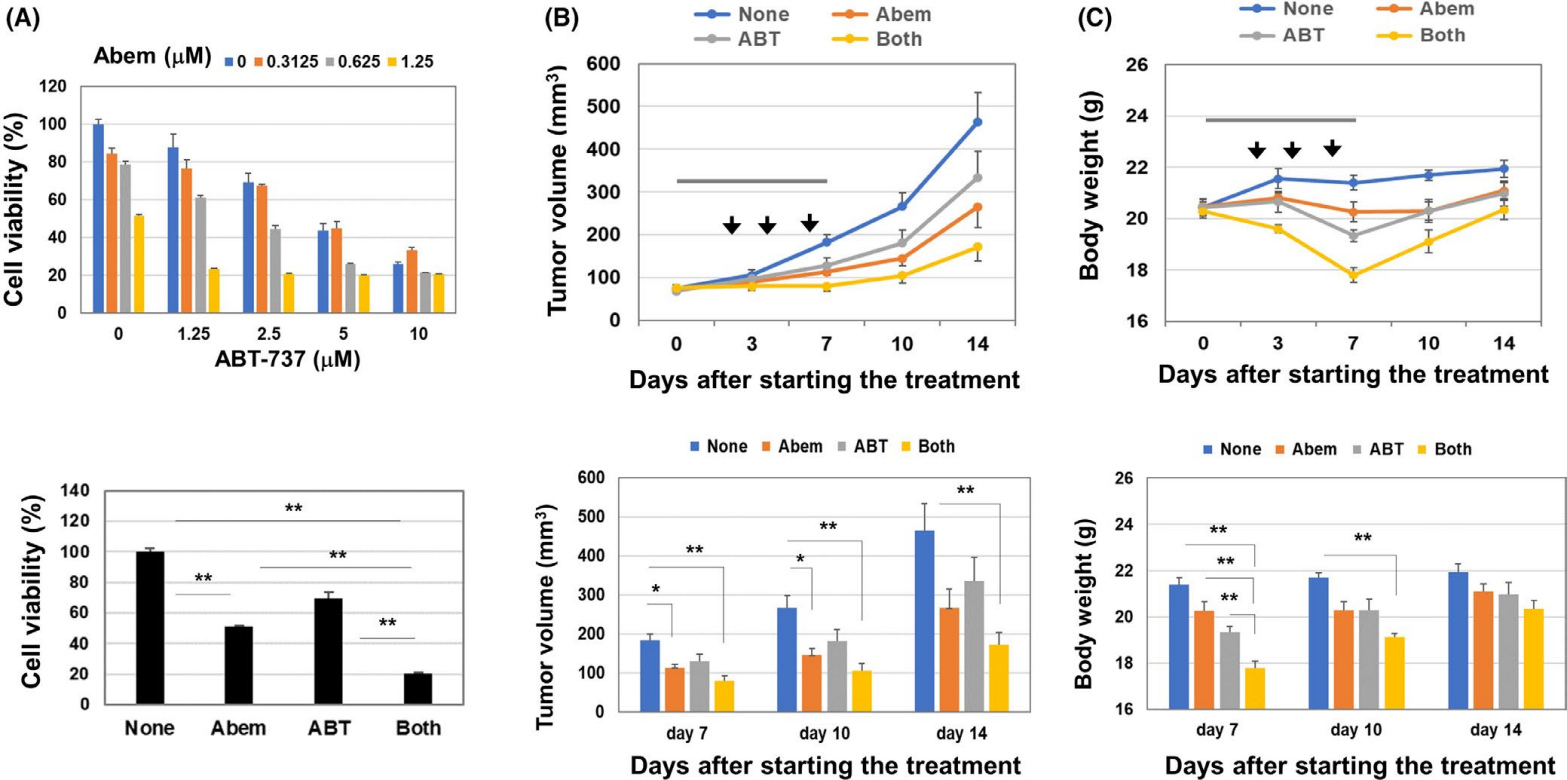
Growth suppression of xenografted MDA‐MB‐231 by abemaciclib and ABT‐737. (A) MDA‐MB‐231 cells were cultured with abemaciclib and/or ABT‐737 for 2 days. Cell viability (%) was assessed by CCR‐8 assay. (Low) Data are the means ± SD of three wells. **p < 0.01. (B, C) BALB nude female mice were injected with MDA‐MD‐231 (3 × 10^6^) cells with Matrigel into the right mammary pad. When the tumor diameter was around 5–6 mm, the mice were divided into four groups. From day 0 to 7 after grouping, mice were administered abemaciclib (50 mg/kg) orally in a volume of 200 µl (gray bar). In some groups, mice were injected intraperitoneally with ABT‐737 (50 mg/kg) on days 2, 4, and 6 after grouping (arrowheads). Tumor size and body weight were measured twice weekly. Each group comprised five or six mice. *p < 0.05, **p < 0.01 (ANOVA with Tukey–Kramer test)

The in vivo effect of abemaciclib and/or ABT‐737 on xenografted MDA‐MB‐231 was examined. Given that abemaciclib and ABT‐263 are orally administered,^10^ we used ABT‐737 because its specificity is identical to ABT‐263 but it can be administered systemically.^12^, ^13^ ABT‐737 showed a similar effect as ABT‐263 when combined with abemaciclib in vitro (Figure 3A). In a xenograft model, abemaciclib alone significantly decreased the tumor volume on days 7 and 10 (*p* < 0.05), and the abemaciclib and ABT‐737 combination further suppressed the tumor volume on days 7, 10, and 14 (*p* < 0.01) (Figure 3B). ABT‐737 alone nonsignificantly suppressed tumor growth. The combination treatment significantly decreased the body weight on day 7 (*p* < 0.01), which was recovered on day 14; that is, 7 days after the final treatment (Figure 3C).

### 3.4 Abemaciclib and ABT‐263 in combination decrease cytoplasmic p21 expression in MDA‐MB‐231 cells

**FIGURE 4:**
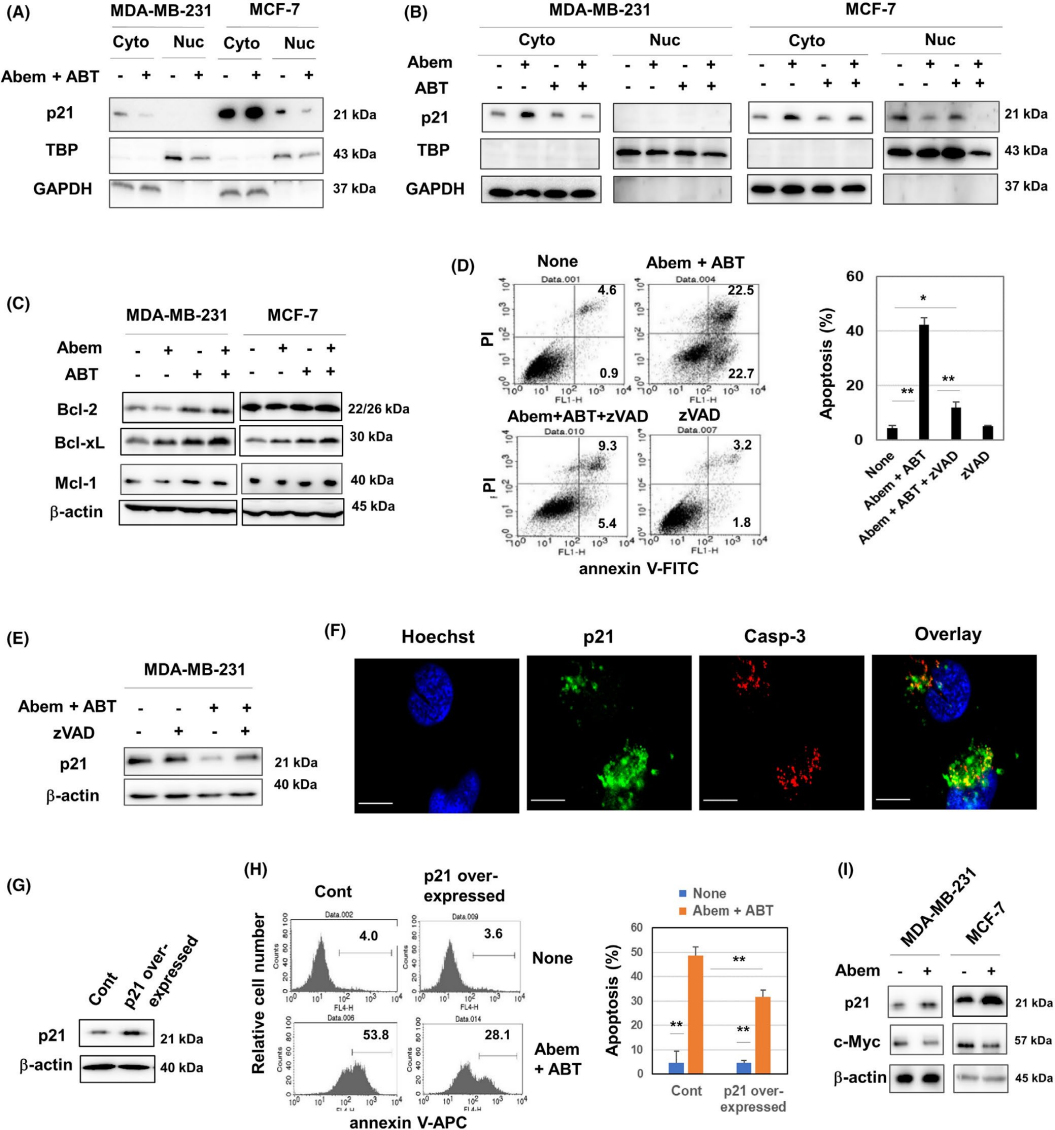
p21 in MDA‐MB‐231 cells treated with abemaciclib and/or ABT‐263. (A, B) Cancer cells were treated with abemaciclib (1.5 µM) and ABT‐263 (1.5 µM) for 2 days. After harvesting, the cytoplasmic and nuclear fractions were separated and subjected to immunoblotting. TBP and GAPDH were used as controls. (C) Immunoblotting was performed similarly using whole lysates. (D) MDA‐MB‐231 cells were treated with abemaciclib (1.5 µM) and ABT‐263 (1.5 µM) with zVAD (10 µM) for 24 h. Flow cytometric analysis was done after staining with annexin V‐FITC and PI. The numbers are proportions of the subsets. (Left) Data of the means ± SD of three cells are shown. *p < 0.05, **p < 0.01. (E) MDA‐MB‐231 cells were treated similarly with zVAD (10 µM) for 24 h and subjected to immunoblotting. (F) Untreated MDA‐MB‐231 cells were cultured with Hoechst 33342 (5 µg/ml) and stained with anti‐p21 and anti‐caspase‐3 antibodies followed by an Alexa 488‐conjugated anti‐rabbit antibody and Cy5‐conjugated anti‐mouse IgG. Confocal imaging reveals nuclei (blue), p21 (green), and caspse‐3 (white). Scale, 10 µm. (G) Control and p21‐overexpressing MDA‐MB‐231 cells were examined for their p21 expression by immunoblotting. (H) Control and p21‐overexpressing MDA‐MB‐231 cells were treated with abemaciclib (1.5 µM) and ABT‐263 (1.5 µM) for 24 h. Flow cytometric analysis was done after staining with annexin V‐APC. Numbers are the proportions of the subset (Left). The means ± SD of four wells are shown. **p < 0.01. (I) Cancer cells were treated with abemaciclib (1.5 µM) for 24 h, and the cell lysates were subjected to immunoblot to examine the expression of p21 and c‐Myc

Abemaciclib induces senescence,^21^ and p16 and p21 play crucial roles in senescence.^22^ Given that MDA‐MB‐231 and MCF‐7 lack p16 (Figure S1), we focused on p21 in the following experiments. The abemaciclib and ABT‐263 combination decreased cytoplasmic p21 expression in MDA‐MB‐231 cells but increased it in MCF‐7 cells (Figure 4A). Nuclear p21 expression was not detected in MDA‐MB‐231 cells, and combination treatment decreased nuclear p21 expression in MCF‐7 cells. As shown in Figure 4B, abemaciclib alone increased cytoplasmic p21 expression in both cell lines as a hallmark of senescence. However, combination treatment decreased and increased cytoplasmic p21 expression in MDA‐MB‐231 and MCF‐7 cells, respectively, compared with the untreated group. Regarding Bcl‐2 family proteins, ABT‐263 showed a tendency to increase the expression of Bcl‐2, Bcl‐xL, and Mcl‐1, irrespective of abemaciclib, in both cell lines (Figure 4C). In addition, zVAD, a pan‐caspase inhibitor, suppressed apoptosis (Figure 4D) and prevented a decrease in p21 expression in treated MDA‐MD‐231 cells (Figure 4E). Considering these results and reports that cytoplasmic p21 binds to and inhibits cleavage of procaspase‐3^23^, ^24^, ^25^, ^26^ we hypothesized that cytoplasmic p21 prevents apoptosis of treatment‐induced senescent MDA‐MB‐231 cells. Confocal imaging revealed that p21 and caspase‐3 were colocalized in the cytoplasm of MDA‐MB‐231 cells (Figure 4F). p21‐overexpressing MDA‐MB‐231 cells exhibited increased resistance to apoptosis compared with the control (Figure 4G,H).

Although CDK4/6, a target of abemaciclib, is a downstream molecule of p21,^22^ abemaciclib increased the expression of the upstream molecule p21 (Figure 4B). On the other hand, it has been reported that c‐Myc represses p21 expression via binding to the *p21* promoter,^27^ and that cellular senescence is linked to tumor regression mediated by c‐Myc inactivation.^28^ To this end, we examined the expression of c‐Myc and found that the abemaciclib treatment decreased the c‐Myc expression in both cell lines, which was in reverse contrast to that of p21. (Figure 4I).

### 3.5 Genetic knockdown of p21 rendered resistant MCF‐7 cells susceptible

**FIGURE 5:**
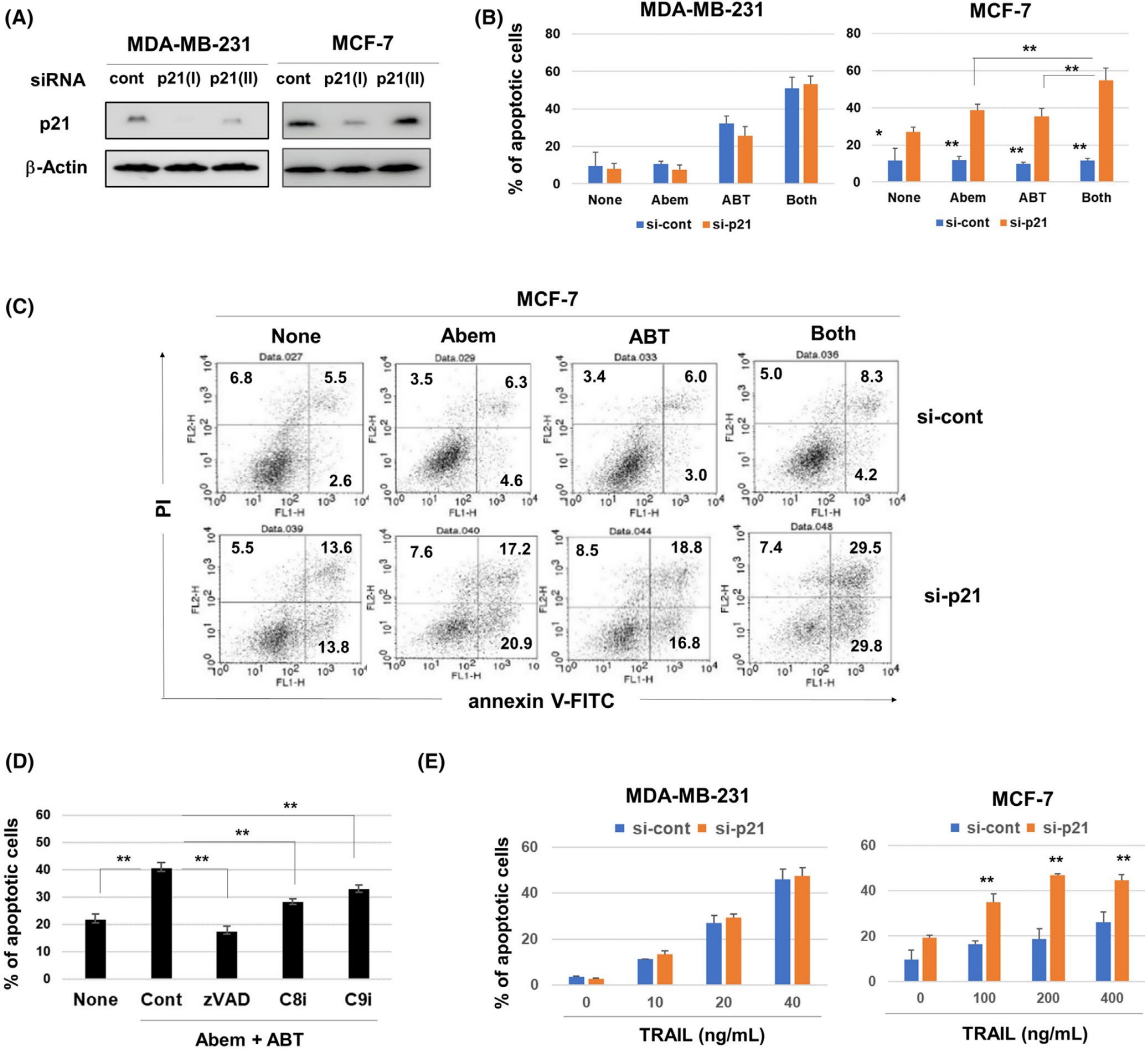
Effect of genetic knockdown of p21 in MCF‐7 cells on their sensitivity to drugs. (A) siRNA‐transfected cancer cells were cultured for 48 h and subjected to immunoblotting. (B) MDA‐MB‐231 and MCF‐7 cells transfected with siRNA p21(I) 2 days prior were treated with abemaciclib (1.5 µM) and ABT‐263 (1.5 µM) for 48 h. After staining with annexin V‐FITC and PI, flow cytometric analysis was performed. Data are the means of three wells. *p < 0.05, **p < 0.01. (C) Representative flow cytometry results; numbers are percentages of the subsets. (D) MDA‐MB‐231 cells were cultured with abemaciclib (1.5 µM) and ABT‐263 (1.5 µM) in the presence of caspase inhibitors (10 µM) for 48 h. Flow cytometric analysis was done after staining with annexin V‐FITC and PI. Data are the means of three wells. **p < 0.01. (E) siRNA p21(I)‐transfected MDA‐MB‐231 and MCF‐7 cells were cultured with TRAIL for 24 h. Flow cytometric analysis was done after staining with annexin V‐FITC and PI. **p < 0.01

We attempted to establish p21‐knockout MDA‐MB‐231 cells by the CRISPR/Cas9 system but were unsuccessful. We therefore tested the effect of siRNA‐mediated knockdown of p21 in breast cancer cells on the sensitivity to abemaciclib and ABT‐263. Because siRNA p21(I) transfection decreased the protein level of p21 in both cell lines more efficiently than siRNA p21(II) (Figure 5A), siRNA p21(I) was used in the following experiments. Although knockdown of p21 showed no effect on apoptosis of treated MDA‐MB‐231 cells, apoptosis was enhanced in p21‐knockdown MCF‐7 cells (Figure 5B). Monotherapy and combination therapy significantly increased the rate of apoptosis of MCF‐7 cells. Representative results of flow cytometry are shown in Figure 5C. The increased apoptosis was suppressed by caspase inhibition (Figure 5D and Figure S2). Immunoblot analysis of a panel of antiapoptotic proteins showed no changes in their expression (Figure S3). We next examined their sensitivity to TRAIL, by which typical caspase‐dependent apoptosis is induced. Knockdown of p21 did not change the rate of TRAIL‐induced apoptosis of MDA‐MB‐231 cells, probably because they were highly sensitive to TRAIL. In contrast, knockdown of p21 increased the sensitivity of MCF‐7 cells to TRAIL (Figure 5E and Figure S4A). siRNA‐mediated knockdown of p21 showed no effect on the surface expression of several TRAIL receptors, such as death receptors (DRs) and decoy receptors (DcRs) (Figure S4B).

We additionally tested the effect of pharmacological inhibition of p21 on the susceptibility of breast cancer cell lines to abemaciclib and/or ABT‐263 in vitro and found that a p21 inhibitor UC2288^29^ sensitized them to ABT‐263 (Figure S5).

### 3.6 Poor prognosis of breast cancer patients with p21^high^ compared to p21^low^

**FIGURE 6:**
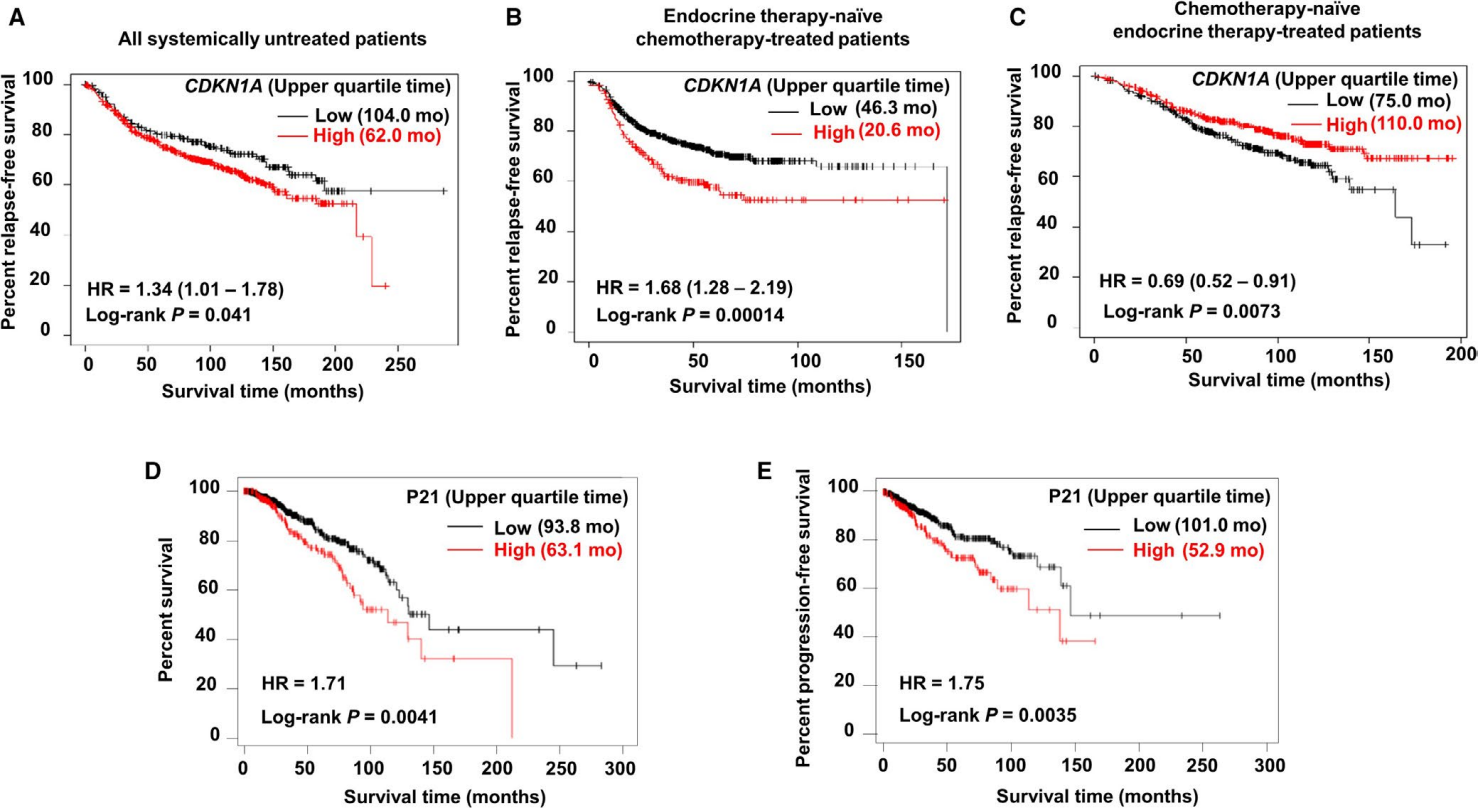
Poor prognosis of breast cancer patients with p21^high^ compared with those with p21^low^. (A, B, C) Kaplan–Meier plotter univariate analysis of survival time in *CDKN1A* mRNA expression in breast cancer. Version 2021 of the database was used for analysis. Outlier array data were excluded for array quality control. Patients were split into low‐ and high‐expression groups based on the optimal cutoff. (D, E) TRGAted was used for survival analysis according to p21 protein level in patients with invasive breast carcinoma. All subtypes of TCGA‐BRCA‐L4 were used in the analysis. Patients were split into low‐ and high‐expression groups based on the optimal cutoff

We finally investigated the influence of p21 expression on the prognosis of breast cancer patients. The Kaplan–Meier plotter^18^ was applied for univariate analysis of survival time according to *CDKN1A* expression in breast cancer. Patients were split into low‐ or high‐ expression groups using the best cutoff. Based on the mRNA level, untreated breast cancer patients with p21^high^ showed a poorer prognosis compared to those with p21^low^ (Figure 6A). Interestingly, chemotherapy‐administered patients with p21^high^ showed a poor prognosis but endocrine therapy‐administered patients with p21^high^ showed a better prognosis (Figure 6B,C). Based on the protein level,^19^ breast cancer patients with p21^high^ exhibited shorter survival (Figure 6D,E).

## 4 DISCUSSION

Abemaciclib, a CDK4/6 inhibitor, induces senescence in cancer cells.^21^ We also observed that abemaciclib can induce senescence‐associated β‐galactosidase, a typical marker of senescence, in MDA‐MB‐231 cells.^17^ Given that p16 and p21 play crucial roles in senescence^22^ and that both cell lines used in this study lack p16, we focused on p21 in the death of treated breast cancer cells because p21 exerts multiple activities in cancer cells.^30^ Abemaciclib monotherapy increased p21 expression in both cell lines, a hallmark of senescence, whereas its combination with ABT‐263 decreased and increased p21 expression in MDA‐MB‐231 cells and MCF‐7 cells, respectively (Figure 4A,B). p21 is a target of p53,^31^ and MDA‐MB‐231 and MCF‐7 cells carry mutant and wild‐type p53, respectively.^32^ In addition, CDK4/6, a target of abemaciclib, is a downstream molecule of p21.^22^ These findings raised the question of why CDK4/6 inhibitors increase the upstream molecule p21. Given that c‐Myc represses p21 expression via binding to the *p21* promoter,^27^ and that cellular senescence has been reported to be responsible for tumor regression upon c‐Myc inactivation.,^28^ we examined the expression of c‐Myc and found that abemaciclib treatment decreased their expression of c‐Myc. (Figure 4I) Presumably, abemaciclib suppressed c‐Myc, resulting in an increase in the p21 level. Given the crucial roles of c‐Myc in cell proliferation,^33^ this possibility is plausible.

Interestingly, inhibition of caspases by zVAD not only prevented combination therapy‐induced apoptosis, but also restored p21 expression in treated MDA‐MB‐231 cells (Figure 4D,E). It has been reported that procaspase‐3 prevents p21 degradation and the procaspase‐3/p21 complex inhibits apoptosis of cancer cells.^23^, ^24^, ^25^, ^26^ Also, caspase‐3 and p21 colocalized in the cytoplasm of MDA‐MB‐231 cells (Figure 4F). Although we tried to confirm the direct binding between caspase‐3 and p21 in MDA‐MB‐231 cells by immunoprecipitation, it was unsuccessful. On the other hand, p21 overexpression rendered MDA‐MB‐231 cells more resistant to the combination treatment (Figure 4H). Therefore, cytoplasmic p21 must protect senescent MDA‐MB‐231 cells from apoptosis.

MCF‐7 cells were relatively resistant to the combination of abemaciclib and ABT‐263 (Figure 2A), which increased p21 expression (Figure 4A). Given that MCF‐7 cells carry wild‐type p53,^30^ this increase must be dependent on p53. Alternatively, genetic knockdown of p21 sensitized MCF‐7 cells to treatment with abemaciclib and ABT‐263 (Figure 5B). Although MCF‐7 cells lack caspase‐3,^20^ the pan‐caspase‐inhibitor zVAD prevented apoptosis of p21‐knockdown MCF‐7 cells in response to either or both abemaciclib or ABT‐263. In addition, p21‐knockdown MCF‐7 cells increased their susceptibility to TRAIL, which induces typical caspase‐dependent apoptosis. Therefore, caspases other than caspase‐3 are implicated in apoptosis of MCF‐7 cells. In addition, p21 inhibits TRAIL‐induced caspase cleavage in SKBR3 human breast cancer cells.^34^ Although the detail mechanisms are unclear, these findings suggest that increased cytoplasmic p21 as a hallmark of senescence protects human breast cancer cells from therapy‐induced apoptosis.

Increased level of p21 has been reported as a biomarker predicting the sensitivity of CDK4/6 inhibitor in estrogen receptor (ER)^+^ breast cancer.^35^ In support of this, ER^+^ p21^high^ MCF‐7 cells showed higher sensitivity to abemaciclib than ER^−^ p21^low^ MDA‐MB‐231 cells (Figure 1A,B). In ER^+^ breast cancer, low expression of p21 leads to promoted cell cycle because p21 failed to inhibit CDK2‐cyclin E complex.^35^ In addition, it is reported that unliganded ERα can inhibit breast cancer cell growth through interaction with p21.^36^ These lines of information provide implication for proliferation of breast cancer cells. On the other hand, in this study, we focused on cancer cell death. We observed that combination treatment‐induced apoptosis was induced in MDA‐MB‐231 cells in association with decreased p21 and that siRNA‐mediated knockdown of p21 sensitized MCF‐7 cells to drug‐induced apoptosis. p21 seems to play different roles in cell growth and cell death of breast cancer cells.

We used ABT‐737 in the xenograft model because it can be administered systemically in vivo, and their combination suppressed the tumor growth significantly (Figure 3B). We measured body weight to evaluate the general condition of treated mice. The combination therapy significantly but transiently decreased body weight, and body‐weight loss was recovered (Figure 3C). These results suggest that combination of CDK4/6 and ABT‐263/ABT‐737 show promise for the therapy of breast cancer.

We determined whether the p21 expression on the prognosis of breast cancer patients by utilizing clinical database.^18^, ^19^ As a result, breast cancer patients with p21^high^ showed a poorer prognosis than those with p21^low^ (Figure 6A,D,E). Interestingly, endocrine therapy‐administered patients with p21^high^ showed a better prognosis but chemotherapy‐administered patients with p21^high^ showed a poor prognosis (Figure 6B,C). These results may have been the results that endocrine therapy exerted effects on cancer proliferation and p21^low^ cancer cells grew rapidly; in contrast, chemotherapy‐induced senescent p21^high^ cancer cells grew slowly but acquired SASP features, leading to cancer progression and metastasis. However, the data should be carefully interpreted because the “endocrine‐treated/chemotherapy‐naive” group might represent patients with ER^+^ luminal breast cancer, while the “endocrine‐naïve/chemotherapy‐treated” group might be patients with ER^−^ or triple‐negative breast cancer (TNBC). The roles of p21 seems to be different between ER^−^ and ER^+^ breast cancer patients.

In conclusion, abemaciclib, a CDK4/6 inhibitor, and ABT‐263/ABT‐737 have therapeutic potential for breast cancer. Cytoplasmic p21, which was increased in CDK4/6 inhibitor‐induced senescent cancer cells, can protect human breast cancer cells from apoptosis. Increased expression of p21 may slow the cell cycle (chemotherapy resistance) and induce apoptosis resistance. Pharmacological inhibition of p21 sensitized breast cancer cells to ABT‐263. These lines of evidence suggest that increased p21 in TIS cancer cells has potential as a target for preventing tumor invasion and metastasis after anticancer therapy.

## CONFLICT OF INTEREST

The authors declare no conflict of interest.

## AUTHOR CONTRIBUTIONS

I.D.K. and M.H. designed the research; I.D.K., H.K., Y.I., A.K., and M.H. performed experiments; I.D.K., R.T., and M.H. analyzed data; I.D.K. and M.H. wrote the paper.

## Supporting information

Fig S1

Fig S2

Fig S3

Fig S4

Fig S5
