# Supplementary figures and images for: Protective role of cytoplasmic p21Cip1/Waf1 in apoptosis of CDK4/6 inhibitor‐induced senescence in breast cancer cells
Source: Cancer Med. 2021 Nov 11;10(24):8988–99. doi: 10.1002/cam4.4410 (PMC8683524; doi:10.1002/cam4.4410)

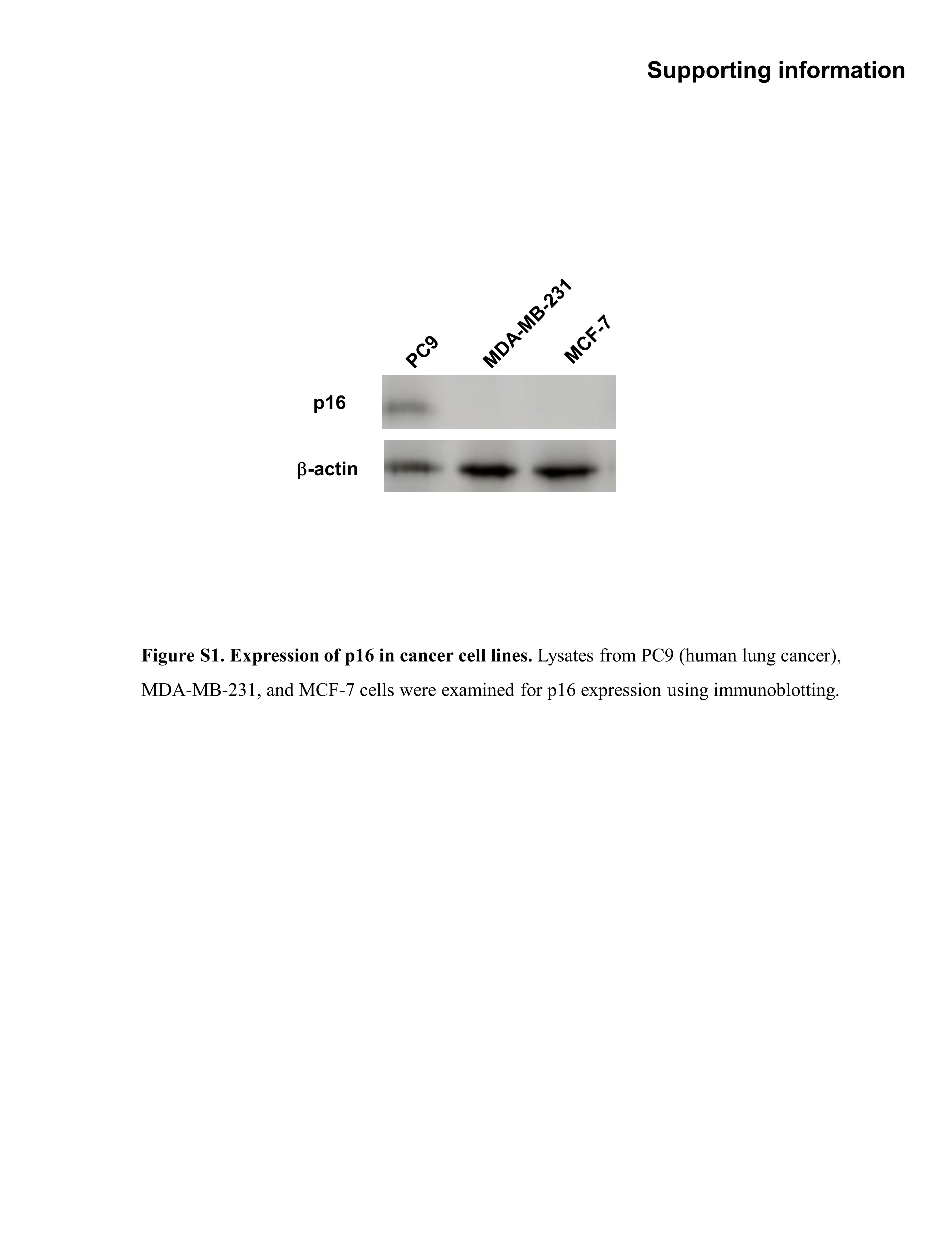

Supplement: Supplementary file 1 — Fig S1 [file CAM4-10-8988-s002.JPG]

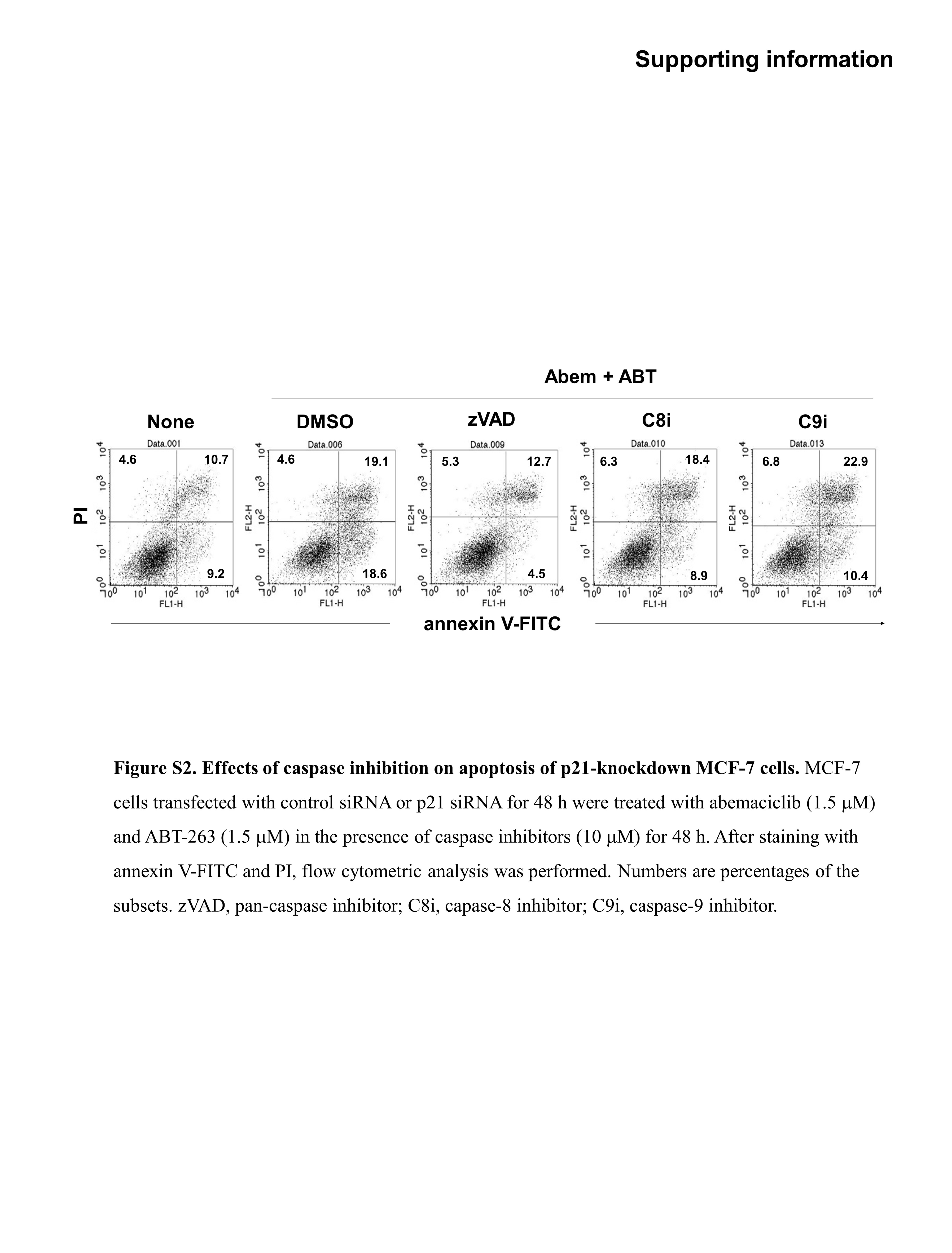

Supplement: Supplementary file 2 — Fig S2 [file CAM4-10-8988-s003.JPG]

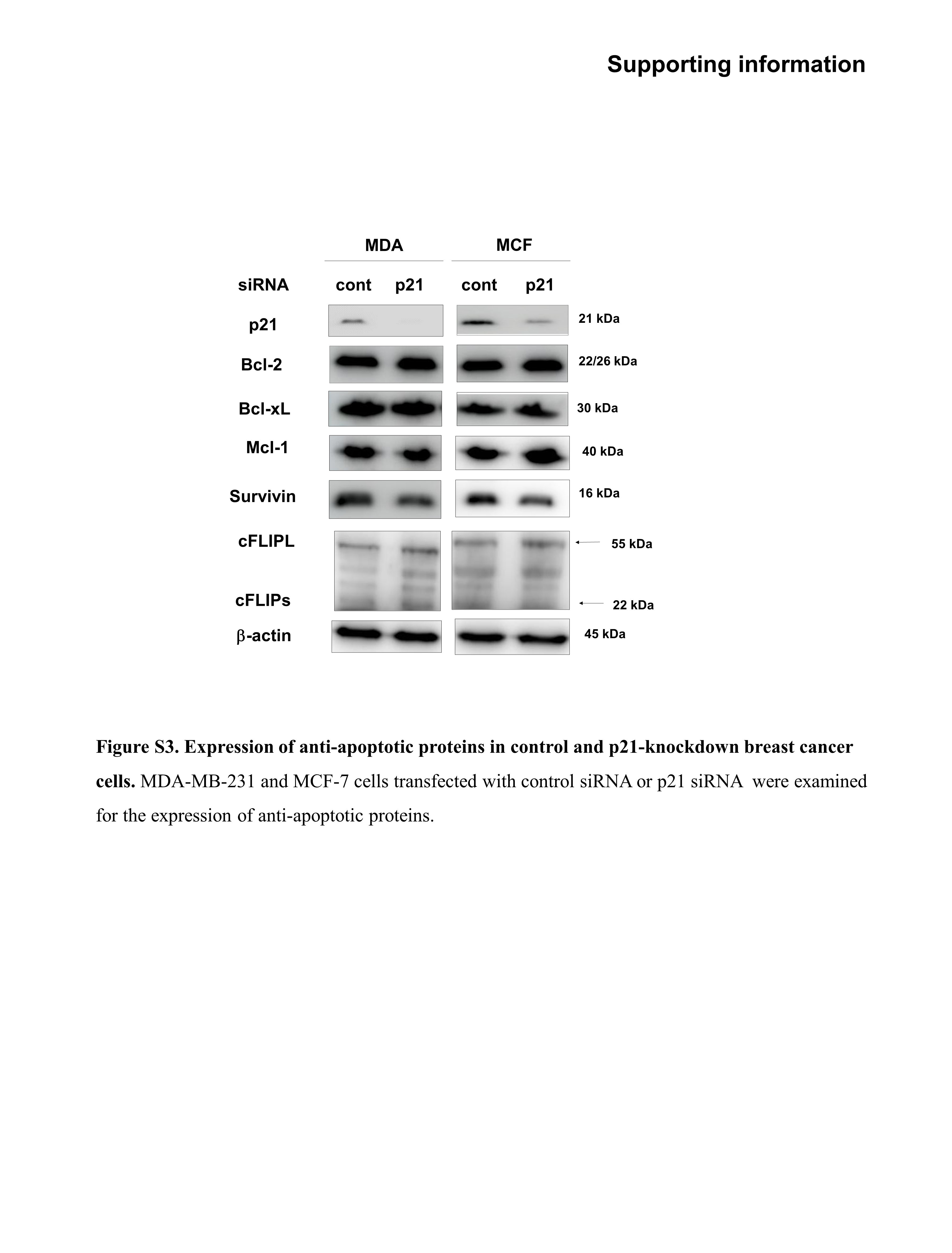

Supplement: Supplementary file 3 — Fig S3 [file CAM4-10-8988-s001.JPG]

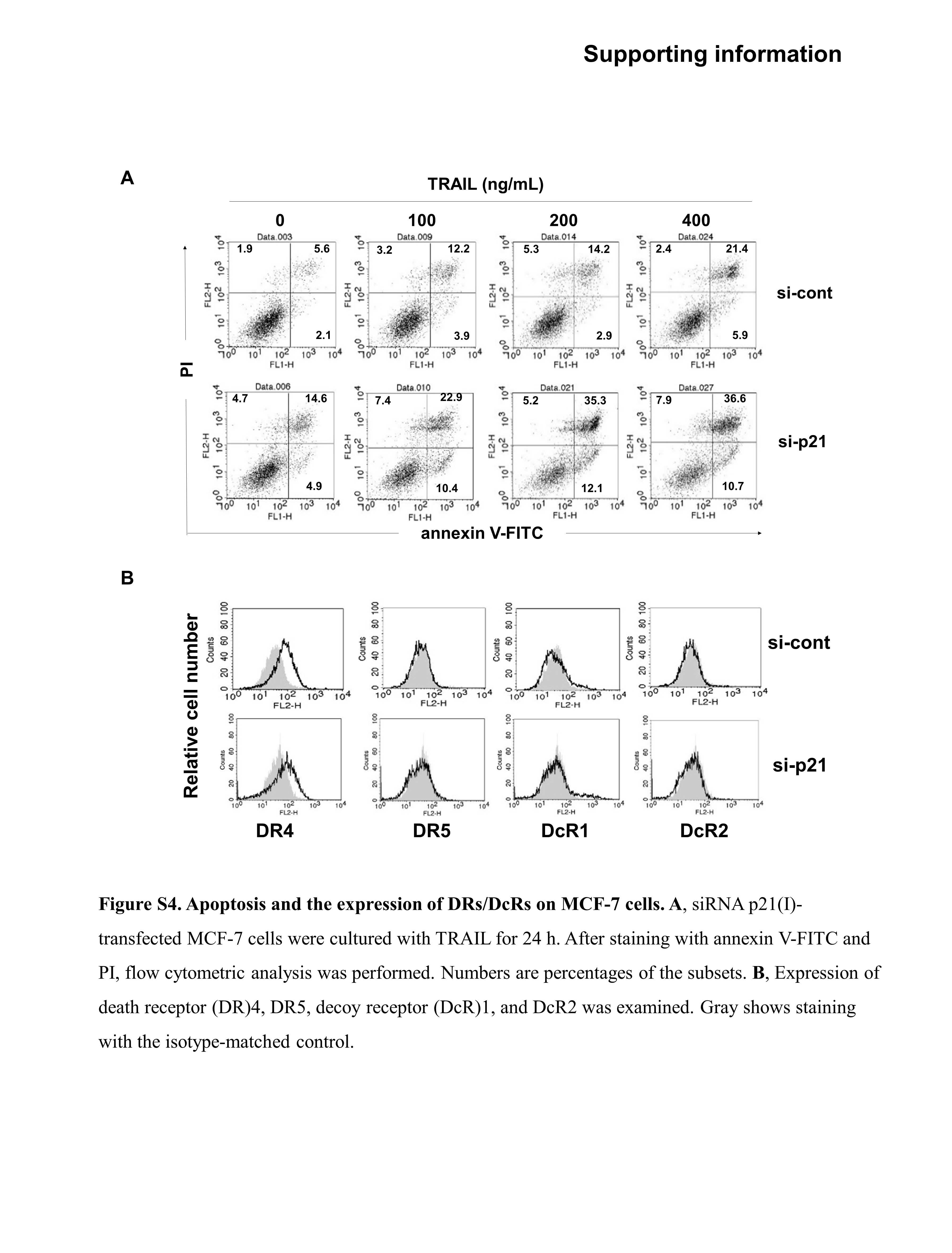

Supplement: Supplementary file 4 — Fig S4 [file CAM4-10-8988-s004.JPG]

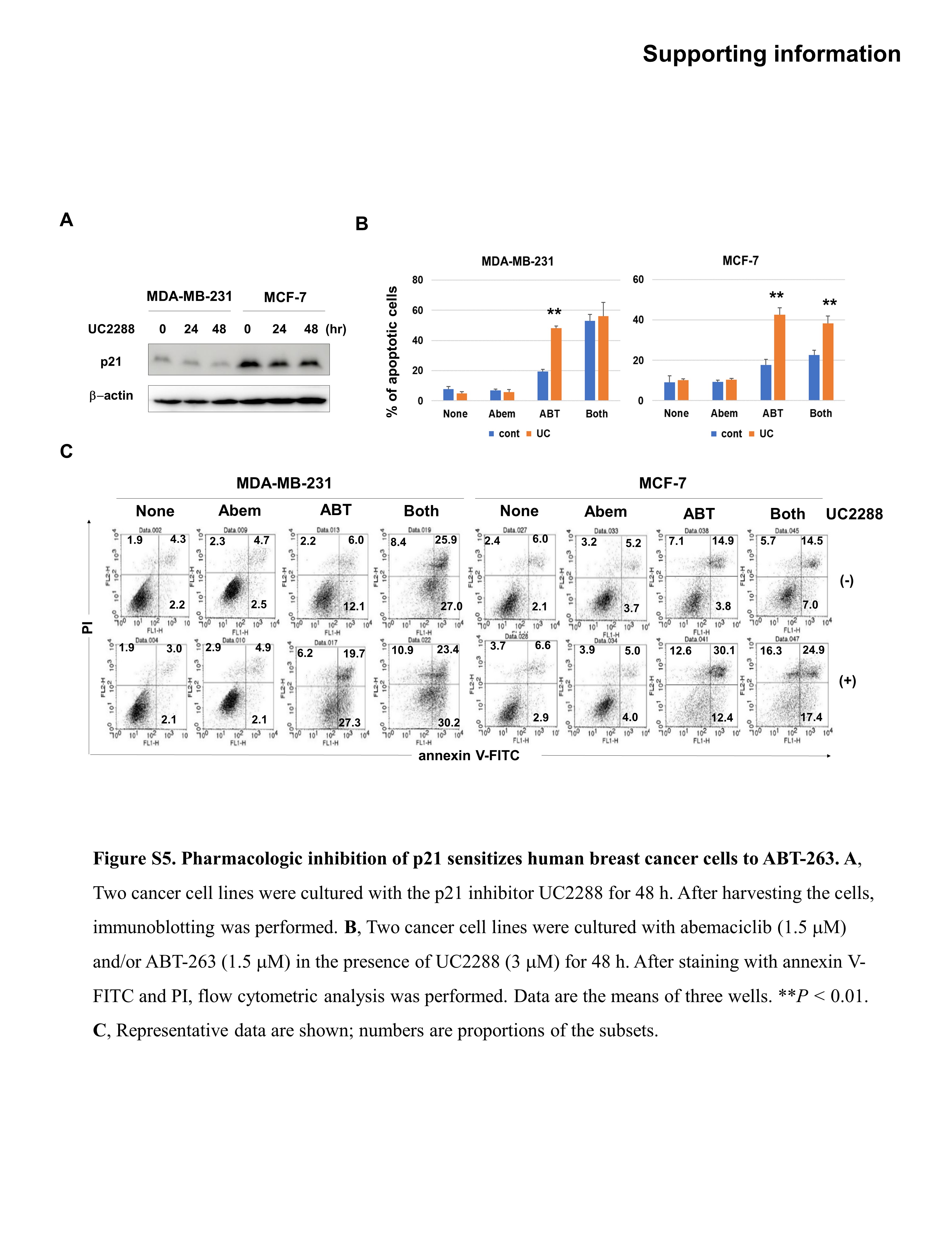

Supplement: Supplementary file 5 — Fig S5 [file CAM4-10-8988-s005.JPG]
